# Spatial-Temporal Relation Enhancement for Speech Emotion Recognition from Acoustic Signals Using Fibonacci Encoding with Diverse Feature Fusion

**DOI:** 10.3390/s26165050

**Published:** 2026-08-09

**Authors:** Shicong Huang, Jinghao Zhang, Jingchao Xu, Qi Zhang, Zihan Li, Songyang Wang, Zirui Qiu, Zijia Xiong, Jintian Liang, Changzeng Fu

**Affiliations:** Sydney Smart Technology College, Northeastern University at Qinhuangdao, Qinhuangdao 066004, China; huangshicong@mails.neu.edu.cn (S.H.); zhangjh1@mails.neu.edu.cn (J.Z.); xujingchao@mails.neu.edu.cn (J.X.); 202212078@stu.neu.edu.cn (Q.Z.); lizihan@mail.neu.edu.cn (Z.L.); wangsongyang@mail.neu.edu.cn (S.W.); qiuzirui@mail.neu.edu.cn (Z.Q.); xiongzijia@mails.neu.edu.cn (Z.X.); jintian.liang@student.uts.edu.au (J.L.)

**Keywords:** speech emotion recognition, acoustic sensing, acoustic signal processing, multi-representation fusion, Fibonacci encoding, positional embedding, Transformer, affective computing

## Abstract

Speech emotion recognition (SER) infers affective states from speech signals, but positional encoding for acoustic tokens remains underexplored in Transformer-based SER. Existing models often reuse encodings designed for text and do not explicitly account for the different sequential and two-dimensional structures of acoustic representations. We propose Fibonacci Position Embedding (FPE) and Fibonacci Target Shutter (FTS). FTS constructs overlapping candidate-index sets over time–frequency token grids, and FPE samples a Fibonacci index and applies its modulo-wrapped, dimension-dependent phase rotation to query and key vectors. The modules are integrated into STRE-Former, which fuses Wav2Vec, log-mel spectrogram, and MFCC representations through asymmetric cross-representation attention with representation-specific positional encodings. We also introduce an implementation-consistent conditional-entropy formulation that quantifies uncertainty in recovering a token location from its sampled positional representation; this quantity characterizes positional ambiguity rather than downstream modeling capacity. Experiments over 64 positional-encoding combinations on IEMOCAP and MELD identify dataset-dependent highest-observed configurations, reaching 74.21% weighted accuracy on IEMOCAP-4, 74.54% on IEMOCAP-6, and 49.44% on MELD. These empirical observations suggest that the relative behavior of positional-encoding strategies may depend on the acoustic representation and evaluation dataset, rather than supporting a single universally optimal scheme.

## 1. Introduction

Speech emotion recognition (SER) aims to automatically infer affective states from speech and is increasingly relevant to affective computing [1], human–computer interaction, and mental health monitoring. As acoustic sensing technology advances, robust SER systems can enable empathetic virtual assistants, assist clinical assessment of mental health conditions, support multimodal interaction systems in education and entertainment, and enhance customer service through emotion-aware dialogue systems.

Early SER methods relied on hand-crafted descriptors such as prosodic statistics and Mel-frequency cepstral coefficients (MFCCs) [2,3]. Deep learning shifted the field toward RNN- and CNN-based models that learn representations directly from spectrograms and frame-level features [4,5]. More recently, self-supervised pre-trained encoders such as Wav2Vec [6] and Transformer architectures [7] have achieved strong performance, while multimodal fusion has further enhanced robustness [8,9]. These advances have substantially improved performance on controlled benchmarks such as IEMOCAP [10] and MELD [11], but significant challenges remain in practical deployment, particularly in handling diverse acoustic conditions, speaker variability, and cross-cultural emotional expressions [12,13].

Despite these advances, the design of positional encodings in SER remains underexplored. Most works adopt absolute positional encoding (APE) or rotary encoding (RoPE) from natural language processing without examining whether one positional treatment is appropriate for all acoustic representations. Spectrograms and MFCCs form time×frequency grids, whereas Wav2Vec embeddings form contextualized temporal sequences. Flattening these structures and applying a single text-derived encoding can discard representation-specific neighborhood information. The resulting gap is not the absence of positional encoding, but the limited systematic investigation of how representation-specific positional strategies behave across heterogeneous acoustic tokenizations.

To study this gap, we propose **Fibonacci Position Embedding (FPE)** and the **Fibonacci Target Shutter (FTS)** for spectro-temporal relation modeling in SER. FTS uses recursive golden-ratio boundaries to construct overlapping candidate-index sets on acoustic feature grids; FPE samples from these sets and converts the selected Fibonacci value into a bounded phase rotation. STRE-Former evaluates this design jointly with conventional encodings across three acoustic representation branches. We analyze the ambiguity introduced by candidate sampling and modulo-wrapped phase mapping without treating it as proof of downstream superiority.

### 1.1. Motivation for Fibonacci-Based Encoding

The Fibonacci design is motivated by two implementation properties rather than by an assumed intrinsic correspondence between the golden ratio and emotion. First, the convergence of adjacent Fibonacci ratios to $\varphi =(1+\sqrt{5})/2$ provides a simple recursive rule for generating nested boundaries on a two-dimensional feature grid. These overlapping regions yield position-dependent candidate-index sets without requiring a learned ordering module.

Second, modular arithmetic maps the sampled Fibonacci values to bounded phases that can be applied as dimension-dependent rotations. The resulting position code is stochastic because FTS samples from each token’s candidate set. Section 3.3 models the resulting chain from token location to sampled index and modulo-wrapped phase. The associated conditional entropy measures positional recovery uncertainty; it does not measure relational richness or imply superiority over deterministic encodings.

For two-dimensional log-mel and MFCC branches, candidate sets are determined by the overlap between token patches and recursively generated regions. Nearby patches may therefore share candidate indices, while each patch can also receive more than one admissible index. This is the structural motivation for FTS; whether it improves recognition is evaluated empirically rather than assumed from the geometry alone.

### 1.2. Contributions

The main contributions of this work are:

1. We propose Fibonacci Position Embedding (FPE) and Fibonacci Target Shutter (FTS), which combine candidate-based Fibonacci index assignment with dimension-dependent phase rotations for spectro-temporal acoustic representations.
2. We introduce the STRE-Former architecture that integrates Wav2Vec, log-mel spectrogram, and MFCC representations via asymmetric co-attention with representation-specific positional encodings.
3. We provide an implementation-consistent conditional-entropy formulation that quantifies positional recovery uncertainty induced by FTS candidate sampling and the modulo-wrapped FPE phase mapping.
4. We conduct systematic ablation studies across 64 positional-encoding combinations on IEMOCAP and MELD, documenting representation- and dataset-dependent highest-observed configurations rather than claiming universal superiority.

## 2. Related Work

### 2.1. Temporal Modeling in Speech Emotion Recognition

Early SER approaches relied on hand-crafted temporal features such as pitch contours, energy trajectories, and MFCCs [2,14]. The advent of deep learning enabled automatic learning of hierarchical representations: RNNs and LSTMs [15] captured sequential dependencies [16], bidirectional LSTMs incorporated future context [17], and CNNs learned local temporal patterns via 1D and 2D convolutions over spectrograms [4,5,18].

The Transformer architecture [7] addressed the limitations of sequential models through self-attention that directly models relationships between all time steps. SpeechFormer [19] introduced hierarchical token semantic learning for SER, while DWFormer [20] proposed dynamic window-based attention with adaptive receptive fields. Self-supervised models such as Wav2Vec 2.0 [6] and HuBERT [21] further advanced the field by learning rich contextual representations from large-scale unlabeled speech, achieving strong fine-tuning performance on SER tasks [22]. The SUPERB benchmark [23] systematically evaluated self-supervised representations across diverse speech tasks including emotion recognition. More recently, large speech models such as Whisper [24] have demonstrated strong generalization across acoustic understanding tasks. Improved attention mechanisms with end-to-end multitask training have also contributed to SER performance gains [25,26].

Recent work has also pursued robust temporal aggregation through capsule, routing, and attention architectures. CENN uses a capsule-enhanced network for robust SER [27], and the sparse temporal-aware capsule network further constrains capsule-based temporal modeling to emphasize informative speech segments [28]. Content-dependent alternatives include pre-attentive processing with adaptive routing [29], while local-to-global feature aggregation [30] and residual attention [31] target complementary acoustic scales or emotion-salient features. These methods modify feature extraction or aggregation topology; they do not directly test how acoustic token coordinates should be encoded. FPE/FTS does not reorder the token tensor; instead, it changes the candidate positional index and phase rotation assigned to each token. It is therefore complementary to capsule routing and residual attention rather than a replacement for them.

These approaches improve temporal modeling through recurrence, hierarchical windows, pre-training, or learned attention, but they generally evaluate an entire architecture rather than isolate how positional encoding interacts with different acoustic representations. Consequently, their reported gains do not establish whether the same positional scheme is appropriate for contextualized waveform sequences and two-dimensional time–frequency features.

### 2.2. Multi-Representation and Conversational Emotion Recognition

Emotion recognition in conversational contexts has attracted significant attention, as emotional expression is inherently contextual and speaker-dependent [9]. Early work modeled inter-speaker and intra-speaker context through memory networks [32]. DialogueRNN [33] used recurrent networks to track speaker states and conversational dynamics, while DialogueGCN [34] modeled inter-speaker relationships through graph convolutions. Multimodal fusion of acoustic, visual, and lexical cues is broadly effective for emotion recognition [8], and cross-modal attention inspired by vision-language pretraining [35] has been adapted for SER.

Within audio-only SER, the Intelligent Fusion Network learns to combine multiple acoustic features with an emphasis on reproducibility and generalization [36]. Such adaptive fusion determines how feature streams contribute to a prediction, whereas the present work asks a different question: how positional information should be assigned within heterogeneous acoustic branches before their interaction. STRE-Former consequently uses representation-specific positional encodings followed by asymmetric co-attention; the fusion mechanism and the positional mechanism remain conceptually distinct.

Data augmentation such as SpecAugment [37] and contrastive learning [38] have further improved robustness against acoustic variability. Knowledge distillation [39] has been explored to transfer representations from large pretrained models to compact SER architectures [40]. Masked prediction pretraining for speech, inspired by BERT [41], has led to HuBERT [21] and related self-supervised frameworks.

These studies address conversational context, augmentation, or cross-modal fusion, whereas the present work uses only audio-derived representations and focuses on positional treatment within and across acoustic branches. We therefore use “multi-representation” for our model and reserve “multimodal” for systems that combine distinct sources such as audio, text, and video.

### 2.3. The Role of Positional Encoding in Speech Emotion Recognition

Positional encoding is crucial for Transformer-based SER because emotional expression unfolds dynamically over time, and the same acoustic pattern may carry different meanings depending on its temporal context. Without adequate positional information, self-attention cannot distinguish sequential order or distance relationships, impeding the separation of transient acoustic fluctuations from sustained emotional trends.

Speech signals present challenges beyond those of text. Spectrograms and MFCCs are inherently 2D time-frequency matrices representing acoustic sensor measurements: when flattened to 1D token sequences via raster-scan ordering, spatially adjacent patches can become distant in the token sequence, weakening explicit local acoustic structure. In a spectrogram of dimensions T×F, a one-step temporal or frequency displacement maps to a different one-dimensional offset, so a single sequence index cannot explicitly distinguish the two axes. Patch-based vision Transformers [42,43] face an analogous 2D locality problem. In audio, the Audio Spectrogram Transformer (AST) represents a spectrogram as a sequence of image-style patches and adds positional information to those tokens [44]; subsequent work has directly compared positional-encoding approaches for audio spectrogram Transformers [45]. These studies establish the relevance of positional design for time–frequency patches, but they do not examine multi-representation SER or stochastic, position-dependent candidate-index assignment.

Existing strategies include Absolute Positional Encoding (APE) [7], Rotary Position Embedding (RoPE) [46], Transformer-XL’s relative encoding [47], relative position representations [48], and Attention with Linear Biases (ALiBi) [49]. APE supplies explicit absolute locations but does not directly encode relative displacement. RoPE rotates query and key pairs so that their interaction depends on relative position, while ALiBi introduces a head-dependent linear distance bias. Learned and adaptive encodings [50,51] provide additional flexibility but introduce data-dependent parameters. These methods were developed mainly for one-dimensional sequences; applying them after flattening a time–frequency grid does not by itself preserve two-dimensional neighborhoods. Our study therefore compares representation-specific combinations rather than assuming that any conventional or Fibonacci-based scheme is universally preferable.

## 3. Fibonacci Position Embedding

Fibonacci Position Embedding (FPE) converts FTS-assigned Fibonacci indices into dimension-dependent complex rotations for acoustic query and key vectors. Its stochastic candidate assignment differs from deterministic APE and RoPE mappings; the consequences of this ambiguity are characterized in Section 3.3 and evaluated separately from recognition performance.

### 3.1. Token Dispersal and Fibonacci Target Shutter

Given an input feature matrix, we divide it into $n_{w}\times n_{h}=N$ patches with dimensions $p_{w}\times p_{h}$. Each patch is mapped to a *d*-dimensional token, yielding $X=(x_{1},x_{2},\ldots ,x_{N})$ where $X\in R^{d\times N}$.

#### 3.1.1. FTS Overview and Intuition

FTS does not reorder the token sequence. It constructs a position-dependent set of admissible Fibonacci indices for each patch on a time–frequency grid and samples one index from that set. Self-attention still receives the tokens in their original flattened order; FTS changes only the phase index applied to each token’s query and key vectors. Overlap among the candidate sets allows nearby or intersecting recursively generated regions to share possible indices, while sampling prevents the assignment from being a deterministic one-to-one position code.

#### 3.1.2. Mathematical Formulation

FTS recursively partitions the patch grid using the inverse golden ratio

(1) $$\tau =\frac{\sqrt{5}-1}{2}.$$

Starting from the full grid bounds, the implementation cycles through left, upper, right, and lower rectangular strips. At each step, it updates the active horizontal or vertical boundary using τ and records the exposed region as $\Gamma_{k}$. The recursion continues until the configured candidate-depth stopping condition is reached; all reported experiments use target_number=10.

For each patch $p_{i}$ with spatial support $\gamma_{i}$, its candidate set contains the indices of all exposed regions intersecting that patch:

(2) $$X_{i}=\left\{k|\gamma_{i}\cap \Gamma_{k}\neq ⌀\right\}.$$

During each positional-encoding construction, the implementation samples $K_{i}$ uniformly from $X_{i}$ using random.choice. The sampled index determines the Fibonacci phase assigned to patch *i*. Equations (9) and (10) give the corresponding probability model used in the uncertainty analysis.

### 3.2. Fibonacci Position Encoding

The position encoding uses a recursively generated, modulo-wrapped Fibonacci sequence:

(3) Fib(x+2)=δ(Fib(x+1)+Fib(x))

where δ(y)=ymod2π keeps the phase argument bounded. Let $f_{K_{p}}$ be the wrapped Fibonacci value selected for token position *p*. For an even attention-head dimension $d_{h}$, the implementation forms $d_{h}/2$ complex dimension pairs with frequencies

(4) $$\theta_{r}={10,000}^{-2r/d_{h}},\;r=0,\ldots ,\frac{d_{h}}{2}-1,$$

and the rotation vector

(5) $$R_{p}^{\mathrm{Fib}}=\left[e^{i\theta_{0}f_{K_{p}}},\ldots ,e^{i\theta_{d_{h}/2-1}f_{K_{p}}}\right].$$

After pairing adjacent real-valued query and key dimensions as complex values, FPE applies the same position-dependent rotation to both:

(6) $${\tilde{q}}_{p}=q_{p}^{C}⊙R_{p}^{\mathrm{Fib}},\;{\tilde{k}}_{p}=k_{p}^{C}⊙R_{p}^{\mathrm{Fib}}.$$

Attention logits are then computed from the real part of the complex query–key product before Softmax. This construction is the operational counterpart of Equation (17).

For the Fib+RoPE variant, the implementation combines the Fibonacci and two-dimensional RoPE rotation matrices before applying them to query and key pairs:

(7) $$R^{\mathrm{hyb}}=\frac{\rho R^{\mathrm{Fib}}+R^{\mathrm{RoPE}}}{\rho +1},$$

where the reported experiments use ρ=1. When enabled, vertical and horizontal flips are independently applied to $R^{\mathrm{Fib}}$ with probability 0.5 before the hybrid combination; both augmentation flags are enabled in the reported configuration.

#### Three-Layer Interpretation

**Recurrence: nonlinear index generation.** The Fibonacci recurrence supplies a nonlinearly varying sequence of index values. We use this sequence as a design choice for generating phase arguments; we do not assume that golden-ratio growth is intrinsically matched to emotional speech.

**Modulo: bounded phase encoding.** The mod2π operation maps Fibonacci values to bounded angles suitable for complex rotations. It can introduce exact or finite-precision phase collisions, but no length-extrapolation guarantee is inferred from this operation.

**Stochastic ambiguity: candidate sampling and phase collisions.** Ambiguity arises first because multiple Fibonacci indices may be admissible for one token and second because modulo wrapping can map different indices to similar phase vectors. Section 3.3 quantifies the resulting position-recovery uncertainty without assuming that greater ambiguity necessarily improves recognition.

### 3.3. Implementation-Consistent Positional Uncertainty Analysis

The implemented FTS/FPE pipeline does not construct idealized modules containing $F_{k}^{2}$ tokens. Instead, FTS produces a position-dependent candidate set, samples a Fibonacci index, wraps the corresponding Fibonacci value modulo 2π, and applies dimension-dependent complex rotations to the query and key vectors. We therefore analyze this realized stochastic mapping directly. All logarithms in this subsection are natural logarithms.

#### 3.3.1. FTS Candidate-Set Construction

Let an input contain *N* tokens and let the original token position be the random variable P∈{1,…,N}. We use a uniform prior over the evaluated positions:

(8) $$\mathrm{Pr}\left(P=p\right)=\frac{1}{N}.$$

For position *p*, FTS recursively partitions the two-dimensional token grid using golden-ratio boundaries. Let $\gamma_{p}$ denote the spatial support of token *p* and $\Gamma_{k}$ the region associated with Fibonacci index *k*. The candidate index set produced by the implementation is

(9) $$X_{p}=\left\{k|\gamma_{p}\cap \Gamma_{k}\neq ⌀\right\}.$$

The implementation samples uniformly from this set. Thus, for the sampled index *K*,

(10) $$q_{pk}=\mathrm{Pr}\left(K=k|P=p\right)=\left\{\begin{array}{cc} |X_{p}{|}^{-1}, & k\in X_{p},\\ 0, & k\notin X_{p}. \end{array}\right.$$

This definition follows the candidate-list construction and uniform random.choice operation used by FTS; it does not impose a Fibonacci-valued module size.

#### 3.3.2. Index-Level Positional Uncertainty

For a fixed token position, the uncertainty of the sampled index is

(11) $$H\left(K|P=p\right)=-\sum_{k}q_{pk}\ln q_{pk}=\ln\left|X_{p}\right|,$$

where the final equality follows from uniform sampling. Averaging over positions gives

(12) $$H\left(K|P\right)=-\frac{1}{N}\sum_{p=1}^{N}\sum_{k}q_{pk}\ln q_{pk}=\frac{1}{N}\sum_{p=1}^{N}\ln\left|X_{p}\right|.$$

Candidate-set entropy alone does not quantify whether a position can be recovered, because disjoint candidate sets may remain position-identifying. The marginal probability of index *k* is

(13) $$q_{k}=\mathrm{Pr}\left(K=k\right)=\frac{1}{N}\sum_{p=1}^{N}q_{pk},\;H\left(K\right)=-\sum_{k}q_{k}\ln q_{k}.$$

Using I(P;K)=H(K)−H(K∣P) and H(P)=lnN, the uncertainty remaining about the token position after observing the sampled index is

(14) H(P∣K)=lnN−H(K)+H(K∣P).

We normalize this quantity as

(15) $$U_{\mathrm{index}}=\frac{H(P|K)}{\ln N},\;0\leq U_{\mathrm{index}}\leq 1.$$

A value of zero means that the sampled index uniquely identifies the position over the evaluated grid, whereas a value of one means that the sampled index is independent of the original position.

#### 3.3.3. Modulo-Wrapped Phase Uncertainty

FPE uses the modulo-wrapped Fibonacci value

(16) $$f_{k}=\mathrm{Fib}\left(k\right)\;\mathrm{mod}\;2\pi .$$

For an even embedding dimension *d*, the *r*-th rotation frequency and the complex phase representation of index *k* are

(17) $$\theta_{r}={10,000}^{-2r/d},\;r=0,\ldots ,\frac{d}{2}-1,\;z\left(k\right)=\left[e^{i\theta_{0}f_{k}},\ldots ,e^{i\theta_{d/2-1}f_{k}}\right].$$

The positional rotation observed for token *p* is therefore the random variable Z=z(K) with $K∼q_{pk}$. To account for finite precision and near-collisions after phase wrapping, we group indices into phase classes using a fixed deterministic clustering rule and the cosine-distance criterion

(18) 1−Re[z(k)Hz(j)]∥z(k)∥2∥z(j)∥2<ε.

Let $B=b_{\varepsilon }\left(K\right)$ denote the resulting phase class. Its position-conditional and marginal probabilities are

(19) $$q_{pb}=\mathrm{Pr}\left(B=b|P=p\right)=\sum_{k:b_{\varepsilon }\left(k\right)=b}q_{pk},\;q_{b}=\frac{1}{N}\sum_{p=1}^{N}q_{pb}.$$

The corresponding entropies and normalized phase-level positional uncertainty are

(20) $$\begin{array}{cc} H(B|P) & =-\frac{1}{N}\sum_{p=1}^{N}\sum_{b}q_{pb}\ln q_{pb}, \end{array}$$

(21) $$\begin{array}{cc} H(B) & =-\sum_{b}q_{b}\ln q_{b}, \end{array}$$

(22) $$\begin{array}{cc} H(P|B) & =\ln N-H(B)+H(B|P), \end{array}$$

(23) $$\begin{array}{cc} U_{\mathrm{phase}} & =\frac{H(P|B)}{\ln N}. \end{array}$$

**Proposition** **1.** *Because P→K→Z→B is a Markov chain and the phase representation and its numerical class are deterministic transformations of the sampled index, the data processing inequality gives*

(24) I(P;B)≤I(P;Z)≤I(P;K),H(P∣B)≥H(P∣Z)≥H(P∣K).

Equality between the index- and phase-level quantities holds when the index-to-phase mapping is injective over the evaluated index range. Thus, modulo wrapping and phase collisions cannot reveal more positional information than the complete sampled index; they can only preserve or increase positional recovery uncertainty.

#### 3.3.4. Expected Positional-Relation Matrix

Conditional entropy measures recoverability, not relational geometry. To describe the geometry induced by the stochastic rotations, we separately define the expected positional-relation matrix

(25) $$G_{pq}=\sum_{a}\sum_{b}q_{pa}q_{qb}\left[\frac{2}{d}\sum_{r=0}^{d/2-1}\cos\;\left(\theta_{r}\left(f_{a}-f_{b}\right)\right)\right].$$

This expectation follows the independent index sampling performed for positions *p* and *q*. For deterministic RoPE, the sampling distribution reduces to a point mass and the corresponding expression uses its standard position-dependent rotation angles. Comparisons among APE, RoPE, and FPE must use the same sequence length, embedding dimension, collision tolerance, and numerical precision. Importantly, $G_{pq}$ characterizes only the geometry induced by positional rotations. It is not the complete attention score, which also depends on the content-dependent query and key vectors.

#### 3.3.5. Scope and Interpretation

The quantities $U_{\mathrm{index}}$ and $U_{\mathrm{phase}}$ measure the uncertainty of recovering a token location from the realized FTS/FPE positional representation. APE and standard RoPE are deterministic position mappings; when they are injective over the evaluated range, their positional recovery uncertainty is zero. FTS/FPE can yield non-zero uncertainty because of candidate sampling and phase collisions. Higher recovery uncertainty must not be interpreted as richer relational information or better SER performance. FPE and FTS are examined empirically through the available component-oriented classification comparisons, while Equation (25) provides a descriptive positional-geometry diagnostic rather than a causal account of attention behavior.

## 4. Proposed STRE-Former Model

### 4.1. Feature Extraction

To comprehensively capture emotional information from acoustic speech signals, we extract three complementary representations that encode different aspects of the waveform and time-frequency sensor measurements.

#### 4.1.1. Wav2Vec 2.0 Features

We employ the pre-trained Wav2Vec2-base-960h model [6] to extract contextualized waveform representations. The model consists of a CNN feature encoder with 7 convolutional layers that downsample the raw waveform (16 kHz sampling rate, 3 s = 48,000 samples) to a sequence of 149 latent representations, followed by a 12-layer Transformer encoder (768-dimensional hidden states, 12 attention heads) that captures long-range dependencies.

For each utterance, we extract the final hidden states from the last transformer layer, yielding $X_{wav}\in R^{149\times 768}$. We use the pre-trained weights without fine-tuning to leverage the rich acoustic representations learned from 960 h of LibriSpeech data. The Wav2Vec2Processor is used to normalize the input waveform before feeding it to the model.

#### 4.1.2. Spectrogram Features

We compute log-mel spectrograms from the Short-Time Fourier Transform (STFT). For frame index τ and frequency-bin index *k*, the magnitude STFT is

(26) $$S\left[\tau ,k\right]=\left|\sum_{n=0}^{N-1}x\left[n+\tau H\right]w\left[n\right]e^{-j2\pi kn/N}\right|,$$

where *n* is the sample offset within a frame, *H* is the hop length, w[n] is the Hamming window, and *N* is the FFT size. Thus, τ, *k*, and *n* respectively denote the frame, frequency bin, and within-frame sample indices.

The extraction parameters are: sampling rate 16,000 Hz; Hamming window; window length 40 ms (640 samples); hop length 10 ms (160 samples); and FFT size 800 points, producing 401 non-redundant linear-frequency STFT bins over 0–8000 Hz. We then apply 200 mel filters and convert the mel power representation to a logarithmic scale. Each input is padded or truncated to 300 frames.

To create a 3-channel representation for the CNN branch, we compute delta and delta-delta features from the log-mel representation:

(27) $$\begin{array}{cc} \Delta \mathrm{spec}(t) & =\mathrm{spec}(t+1)-\mathrm{spec}(t-1) \end{array}$$

(28) $$\begin{array}{cc} \Delta^{2}\mathrm{spec}\left(t\right) & =\Delta \mathrm{spec}(t+1)-\Delta \mathrm{spec}(t-1) \end{array}$$

The resulting spectrogram $X_{spec}\in R^{3\times 200\times 300}$ contains the original log-mel spectrogram, delta, and delta-delta features as three channels.

#### 4.1.3. MFCC Features

Mel-Frequency Cepstral Coefficients (MFCCs) capture the timbral texture of speech by mimicking human auditory perception. We compute MFCCs using the Discrete Cosine Transform (DCT) applied to log mel-filterbank energies:

(29) $$\mathrm{MFCC}\left[n\right]=\sum_{m=1}^{M}\log\left(E_{m}\right)\cos\left[n\left(m-\frac{1}{2}\right)\frac{\pi }{M}\right]$$

where $E_{m}$ is the energy in the *m*-th mel filter bank, and *M* is the number of filters.

The extraction parameters are: number of MFCC coefficients 40; hop length 160 samples (10 ms); HTK formula enabled for compatibility; time frames 300 (matching spectrogram).

The extracted MFCCs form a matrix $X_{MFCC}\in R^{300\times 40}$, which is normalized using L2 normalization along the feature dimension to ensure stable training.

#### 4.1.4. Complementary Nature of Features

The three acoustic representations provide complementary views of the same waveform: **Wav2Vec** supplies contextualized acoustic representations learned through self-supervised pre-training; the **log-mel spectrogram** retains localized time–frequency energy, harmonic structure, and temporal changes; and **MFCC** compactly represents the spectral envelope and vocal-tract-related characteristics.

This multi-view representation enables the model to leverage both low-level acoustic details and high-level semantic information for robust emotion recognition.

### 4.2. Tokenization and STRE-Former Architecture

**Figure 1:**
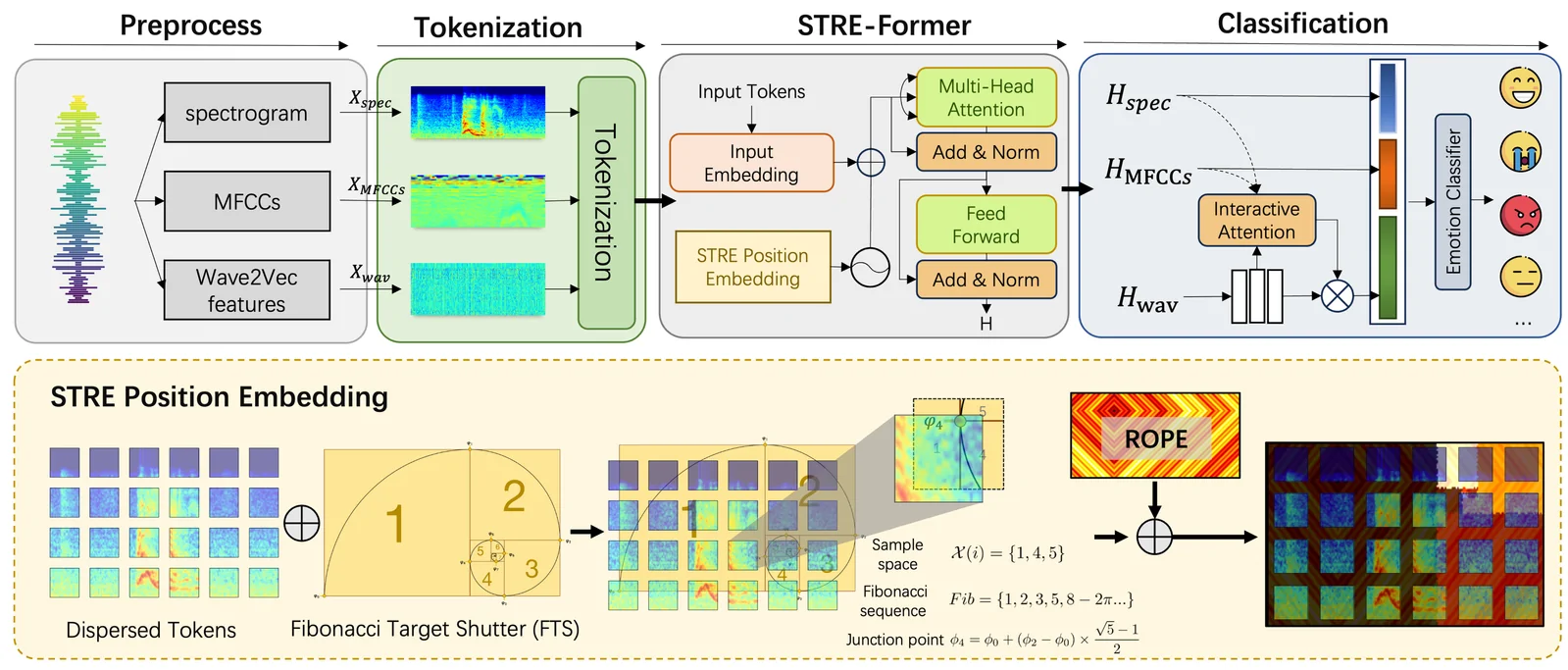
Architecture of the proposed STRE-Former. Three representations derived from the same audio signal (Wav2Vec 2.0, log-mel spectrogram, and MFCC) are tokenized and processed by branch-specific transformer encoders. FTS assigns candidate-based Fibonacci phase indices to two-dimensional log-mel/MFCC patches without changing their flattened token order. An asymmetric cross-representation co-attention module fuses the three branches, and an MLP head predicts the emotion category.

Each acoustic feature undergoes tokenization into patch-based representations $X_{wav}^{\prime },X_{spec}^{\prime }$, and $X_{MFCC}^{\prime }$. STRE-Former then processes each representation with a branch-specific positional encoding and self-attention module. For transparency, the evaluated configurations are not identical in attention-head count: APE, RoPE, and ALiBi branches use four heads, whereas Fib and Fib+RoPE branches use one. Consequently, comparisons involving these branches are comparisons between complete configurations rather than strictly isolated positional-encoding effects. For each branch, the attention output is $H=\Psi (X^{\prime })$, and feed-forward layers with ReLU produce $H_{wav}$, $H_{spec}$, and $H_{MFCC}$. The overall architecture is illustrated in Figure 1.

### 4.3. Cross-Modal Co-Attention and Feature Fusion

To integrate complementary information across representation branches, we design an asymmetric co-attention mechanism. We adopt this structure as a practical fusion choice motivated by the different roles of frequency-domain and contextualized waveform representations. Log-mel and MFCC features provide spectral information, while Wav2Vec embeddings provide contextualized representations from pre-training. Frequency-domain features are used as queries over the Wav2Vec sequence; this is an architectural design choice rather than evidence that one representation is intrinsically more informative.

Log-mel spectrograms retain localized frequency-domain dynamics, while MFCCs compress the spectral envelope into perceptually motivated coefficients. Their concatenation forms a joint query for the Wav2Vec branch. We use frequency-to-waveform rather than bidirectional attention to limit computation; the contribution of this asymmetry is not isolated in the present experiments.

#### 4.3.1. Co-Attention Mechanism

We concatenate the spectrogram and MFCC representations to form a joint query:

(30) $$q=\left[h_{spec};h_{MFCC}\right]\in R^{256}$$

This query is projected and used to compute attention weights over the Wav2Vec sequence:

(31) $$\begin{array}{cc} q^{\prime } & =\mathrm{ReLU}({\mathrm{Linear}}_{256\to d_{q}}\left(\mathrm{Dropout}\left(q\right)\right)) \end{array}$$

(32) $$\begin{array}{cc} q^{\prime \prime } & =\mathrm{Reshape}\left(q^{\prime },\left[1,d_{q}\right]\right)\in R^{1\times d_{q}} \end{array}$$

where $d_{q}$ depends on the positional encoding used in the Wav2Vec branch. To match dimensions, we apply a branch-specific adjustment layer:

(33) $$q_{adj}={\mathrm{Linear}}_{d_{q}\to d_{wav}}\left(q^{\prime \prime }\right)$$

The attention-weighted Wav2Vec representation is computed via matrix multiplication:

(34) $$\begin{array}{cc} h_{wav}^{att} & =q_{adj}\cdot H_{wav}\in R^{1\times 768} \end{array}$$

(35) $$\begin{array}{cc} h_{wav}^{flat} & =\mathrm{Reshape}(h_{wav}^{att},\left[768\right]) \end{array}$$

(36) $$\begin{array}{cc} h_{wav} & =\mathrm{ReLU}({\mathrm{Linear}}_{768\to 128}\left(\mathrm{Dropout}\left(h_{wav}^{flat}\right)\right)) \end{array}$$

#### 4.3.2. Feature Fusion

The three branch-specific representations are concatenated:

(37) $$h_{fused}=\left[h_{spec};h_{MFCC};h_{wav}\right]\in R^{384}$$

### 4.4. Emotion Classification and Training

#### 4.4.1. Classification Head

The fused feature vector is fed through a multi-layer perceptron (MLP), which returns un-normalized class logits:

(38) $$\begin{array}{cc} h_{1} & =\mathrm{ReLU}({\mathrm{Linear}}_{384\to 128}\left(\mathrm{Dropout}\left(h_{fused}\right)\right)) \end{array}$$

(39) $$\begin{array}{cc} h_{2} & =\mathrm{ReLU}({\mathrm{Linear}}_{128\to 128}\left(\mathrm{Dropout}\left(h_{1}\right)\right)) \end{array}$$

(40) $$\begin{array}{cc} s & ={\mathrm{Linear}}_{128\to C}\left(h_{2}\right), \end{array}$$

where *C* is the number of emotion classes (4 for IEMOCAP-4, 6 for IEMOCAP-6, and 7 for MELD). Dropout with probability 0.1 is applied in the MLP. Log-probabilities $\ell_{i,c}=\log\mathrm{Softmax}{\left(s_{i}\right)}_{c}$ are computed inside the training criterion; at inference, the predicted class is $\arg\max_{c}s_{i,c}$. Thus, Softmax is not an additional trainable output layer.

#### 4.4.2. Loss Function

The reported implementation uses a log-probability-based classification criterion on the logits. For one-hot label $y_{i,c}$ and log-probability $\ell_{i,c}$, the implemented objective is

(41) $$L_{\mathrm{cls}}=-\frac{\alpha }{BC}\sum_{i=1}^{B}\sum_{c=1}^{C}{\left(1-y_{i,c}\ell_{i,c}\right)}^{\gamma }\ell_{i,c},$$

with α=1 and γ=2.5. This equation directly documents the criterion used in the reported experiments rather than assigning it a standard loss-function label. Its factor ${\left(1-y_{i,c}\ell_{i,c}\right)}^{\gamma }$ differs from the standard focal-loss factor ${\left(1-p_{t}\right)}^{\gamma }$, and the resulting terms are averaged over all classes. No class weights, learning-rate scheduler, or separate $\lambda {∥\Theta ∥}_{2}^{2}$ penalty are applied.

## 5. Experimental Setup

### 5.1. Datasets

We evaluate on IEMOCAP and MELD because they provide complementary acoustic and conversational conditions. IEMOCAP contains controlled dyadic interactions with comparatively clear utterance boundaries, whereas MELD contains natural multi-party conversations, more speakers and transitions, and a markedly imbalanced label distribution. Together they test the model in both controlled two-speaker and more complex conversational settings.

#### 5.1.1. IEMOCAP Dataset

The Interactive Emotional Dyadic Motion Capture (IEMOCAP) database [10] contains approximately 12 h of audiovisual recordings from 10 professional actors (5 male and 5 female) in five dyadic sessions with scripted and spontaneous interactions. In the standard four-class setting, excitement is merged with happiness, giving 5531 utterances across anger, happiness/excitement, sadness, and neutrality. The six-class setting treats happiness and excitement separately and additionally includes frustration. Each utterance is annotated by multiple evaluators, and the majority label is used as ground truth. Audio is sampled at 16 kHz, and utterances are padded, truncated, or segmented into fixed-length inputs during preprocessing.

#### 5.1.2. MELD Dataset

The Multimodal EmotionLines Dataset (MELD) [11] is derived from the television series “Friends” and provides natural multi-party conversations with seven emotion labels: anger, disgust, fear, joy, neutral, sadness, and surprise. Although MELD contains audio, text, and video, this study uses only its audio track. The official train/development/test splits contain 9989, 1109, and 2610 utterances, respectively. The test split is strongly imbalanced, ranging from 1256 neutral utterances to 50 fear utterances. This distribution, together with conversational speaker changes and natural recording variability, makes MELD complementary to the more controlled IEMOCAP setting.

### 5.2. Evaluation Metrics

We report **Weighted Accuracy (WA)**, i.e., overall sample accuracy; **Unweighted Accuracy (UA)**, the unweighted mean of class-wise recalls; **Weighted F1 (WF1)**, the support-weighted mean of class-wise F1 scores; and **Unweighted F1 (UWF1)**, the macro-averaged F1 score. Segment-level predictions are averaged within each utterance before utterance-level evaluation. All reported values are rounded to two decimal places for presentation consistency.

### 5.3. Implementation Details

All experiments are conducted on an NVIDIA GeForce RTX 4090 (NVIDIA Corporation, Santa Clara, CA, USA) using PyTorch 2.1.2 (Linux Foundation, San Francisco, CA, USA) and librosa 0.10.2. The model is trained for up to 500 epochs with AdamW, batch size 16, dropout 0.1, and early-stopping patience 100. The optimizer is instantiated without an explicit weight_decay argument and therefore uses the PyTorch 2.1.2 AdamW default of $10^{-2}$; no second L2 penalty is applied in the loss. No learning-rate scheduler or class weighting is used. The configuration used for the reported experiments specifies one training repeat with RANDOM_SEED = 66. Because only one seed is reported, small score differences should not be interpreted as statistically stable.

The experiments are conducted in two stages. First, all 64 configurations are evaluated with a learning rate of $1\times 10^{-4}$ to describe broad configuration-level trends and identify candidates for refinement. Second, the highest-observed candidate and the APE and ALiBi baselines are evaluated at $1\times 10^{-4}$ and $1\times 10^{-5}$. RoPE is included in the common-budget first-stage screening but does not receive the same second-stage learning-rate refinement budget. Accordingly, first-stage results are used only for broad configuration-level observations, and comparisons between an unrefined RoPE score and a second-stage-refined maximum are not interpreted as strictly optimization-budget-matched rankings. The original 64-configuration analysis was designed as exploratory configuration screening rather than as a fully nested model-selection experiment. We therefore do not interpret the highest-observed configuration as an unbiased estimate of a universally optimal architecture. The revised analysis explicitly identifies selection variability and treats small differences among shortlisted configurations as descriptive observations.

#### Evaluation Protocol

We follow the widely adopted leave-one-session-out (LOSO) protocol on IEMOCAP. Specifically, each fold holds out one session for testing and uses the remaining sessions for training and validation, and the reported results are averaged across all folds. The reported configuration search is interpreted as exploratory screening rather than an unbiased nested model-selection evaluation.

## 6. Results and Analysis

### 6.1. Performance Comparison

Table 1 reports prior results alongside STRE-Former. The highest-observed refined STRE-Former triplets are (Fib, Fib+RoPE, Fib+RoPE) on IEMOCAP-4, (Fib, Fib+RoPE, RoPE) on IEMOCAP-6, and (APE, Fib+RoPE, Fib+RoPE) on MELD. Their respective WA values are 74.21%, 74.54%, and 49.44%. Under the reported protocols, the IEMOCAP scores are higher than the listed audio-only baselines, while the MELD score remains below SpeechFormer++ in WA. SpeechCueLLM [52] and AA-SLLM [53] are included only as contextual references because they use text or speech-language-model-scale pre-training beyond our audio-only setting. Cross-study rankings remain tentative because preprocessing, feature extraction, capacity, and evaluation protocols are not controlled.

From a computational perspective, we compare the complete highest-observed configuration (Fib, Fib+RoPE, Fib+RoPE) against complete APE and ALiBi baselines. All measurements are conducted on an NVIDIA GeForce RTX 4090 with batch size 16, using 50 warm-up iterations followed by 200 measurement runs. The configurations differ in attention-head count, so Table 2 should not be interpreted as an isolated measurement of positional-encoding overhead.

**Figure 2:**
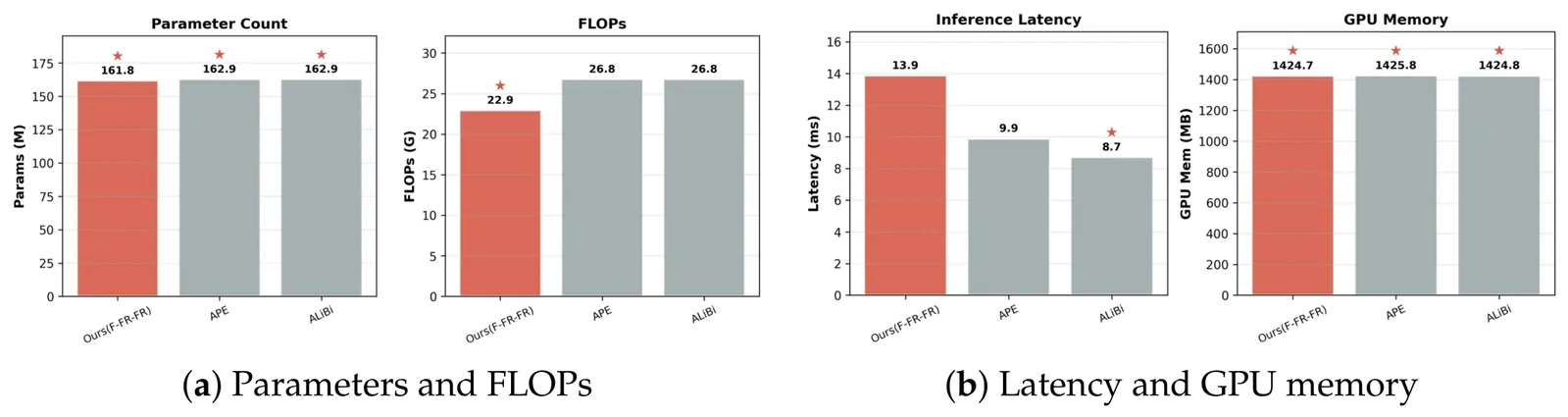
Configuration-level complexity comparison. The orange/red bars denote the proposed Fibonacci configuration (Ours, F-FR-FR), and the gray bars denote the conventional APE and ALiBi baselines. Red stars indicate the optimal or near-optimal value for each metric. The Fibonacci configuration has a lower measured parameter count and FLOPs but higher latency than the four-head APE and ALiBi configurations; GPU memory is comparable. Head counts and other implementation differences are not controlled in this figure.

As shown in Table 2, the evaluated Fibonacci configuration has fewer parameters (161.79 M vs. 162.90 M) and lower FLOPs (22.94 G vs. 26.78 G), but higher latency (13.87 ms vs. 9.89/8.71 ms); GPU memory is comparable (≈1425 MB). These differences reflect the complete implementations, including the one-head versus four-head design and candidate-based phase construction. The measurements do not isolate the computational effect of FTS or FPE alone. Figure 2 visualizes the observed configuration-level trade-offs.

### 6.2. Ablation Study: Positional Encoding Combinations

**Figure 3:**
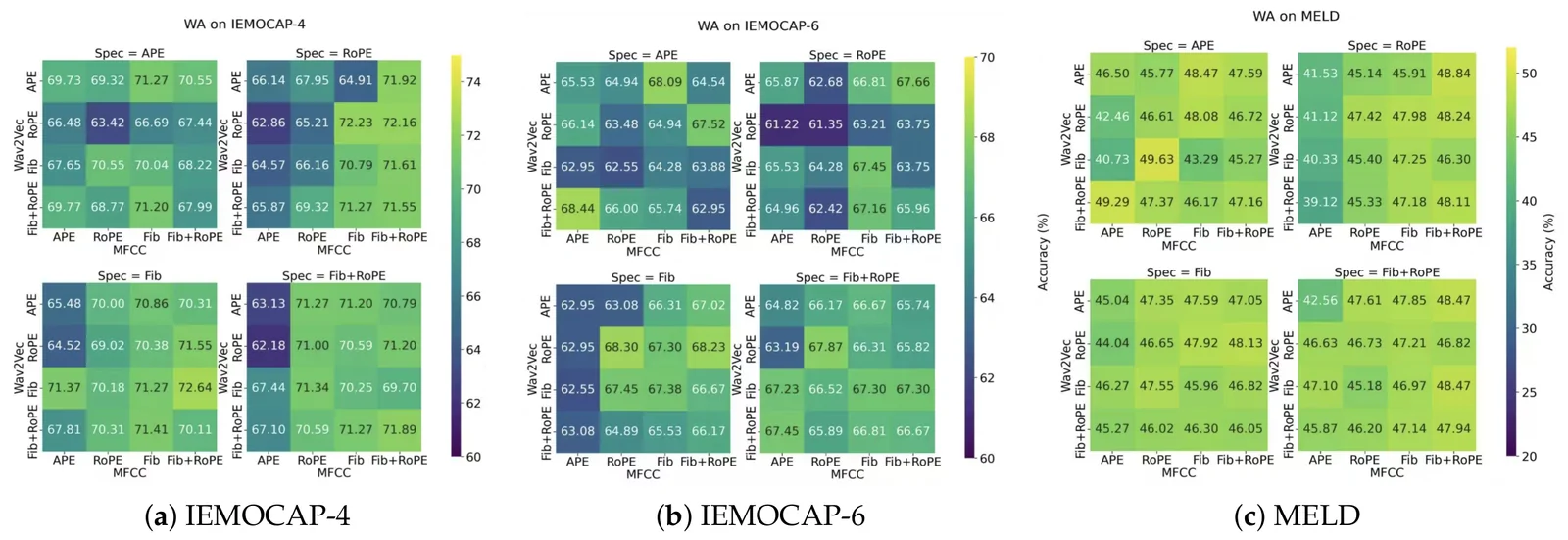
Visualization of weighted accuracy (WA) across 64 positional encoding combinations on (**a**) IEMOCAP-4, (**b**) IEMOCAP-6, and (**c**) MELD. Each subplot represents different spectrogram encodings (APE, RoPE, Fib, Fib+RoPE), with rows indicating Wav2Vec encodings and columns showing MFCC encodings. Brighter colors indicate higher performance in each figure.

To examine positional-encoding configurations, we evaluate 64 combinations across the log-mel, MFCC, and Wav2Vec branches. Each triplet (Spectrogram, MFCC, Wav2Vec) uses APE, RoPE, Fib, or Fib+RoPE in each position. All 64 configurations use a learning rate of $1\times 10^{-4}$ in this exploratory stage. This controls the learning rate but not all architectural factors, because conventional and Fibonacci branches differ in attention-head count. Figure 3 therefore reports complete-configuration results rather than isolated positional-encoding effects.

The observed scores vary across representation branches and datasets, and no configuration is highest in every setting. Because the configurations also differ in head count and are selected from a large search space, these results establish empirical associations rather than a causal effect of positional encoding alone. They motivate further capacity-matched evaluation of representation-specific positional strategies.

The first stage uses a common learning rate ($1\times 10^{-4}$), batch size 16, up to 500 epochs, and early stopping to identify broad trends. The second stage evaluates the shortlisted configurations and the APE and ALiBi baselines at $1\times 10^{-4}$ and $1\times 10^{-5}$; RoPE does not receive an identical second-stage refinement budget. The heatmaps show common-budget first-stage results before learning-rate refinement. We use these first-stage results only for broad configuration-level observations and do not rank an unrefined RoPE result directly against a second-stage-refined maximum as if their optimization budgets were matched. Since many configurations are screened and optimization exposure is not fully matched across encoding families, small differences among the highest values should not be interpreted as stable or strictly budget-matched rankings.

Several descriptive patterns appear in the first-stage results. Fib-Fib-Fib obtains 72.15% WA on IEMOCAP-4, 72.87% on IEMOCAP-6, and 47.89% on MELD. Fib+RoPE in the MFCC branch appears in each highest-observed refined triplet, whereas the selected log-mel and Wav2Vec encodings vary by dataset. These observations are hypotheses for follow-up capacity-matched and multi-seed experiments, not evidence that a particular encoding has an intrinsic advantage for one acoustic representation.

Figure 3 visualizes WA across the 64 complete configurations. APE-APE-APE obtains 69.73%, 65.53%, and 46.50% WA on IEMOCAP-4, IEMOCAP-6, and MELD, respectively; RoPE-RoPE-RoPE obtains 65.21%, 61.35%, and 47.42%. These values provide reference points within the search but do not isolate the encoding from the accompanying architecture.

Figure 3 and the refined results show that the highest-observed triplet differs among datasets. IEMOCAP-4 selects (Fib, Fib+RoPE, Fib+RoPE), IEMOCAP-6 selects (Fib, Fib+RoPE, RoPE), and MELD selects (APE, Fib+RoPE, Fib+RoPE). Fib+RoPE appears in the MFCC branch of all three selected triplets, while the log-mel and Wav2Vec choices vary. This is a descriptive pattern from the evaluated search and does not establish a universal representation–encoding rule. Table 3 reports the selected configurations and metrics.

Table 4 reports the available three-state component comparison. “FPE-only” retains Fibonacci phase rotation but disables FTS candidate sampling by using sequential Fibonacci indices. “FPE+FTS” uses candidate-based sampling. An FTS-only state is not separately identifiable in the implementation because FTS outputs an index that is consumed by FPE; disabling FPE removes the downstream path through which the FTS index acts.

Within the available runs, candidate-based FTS assignment is associated with increases of 4.10 and 7.24 percentage points over FPE-only on the two IEMOCAP settings and 0.37 points on MELD. These are single-run configuration differences; without repeated capacity-matched runs, they document observed configuration-level differences rather than a stable causal effect.

### 6.3. MELD Class Distribution and Restored-Checkpoint Error Analysis

We complement the aggregate MELD result with the official emotion-label distribution and an error analysis from the retained checkpoint. These two sources use different counting bases and are therefore reported separately: Table 5 describes the 2610 raw utterances in the official test split, whereas Table 6 and Table 7 describe the 3808 evaluation samples processed by the restored inference pipeline.

**Figure 4:**
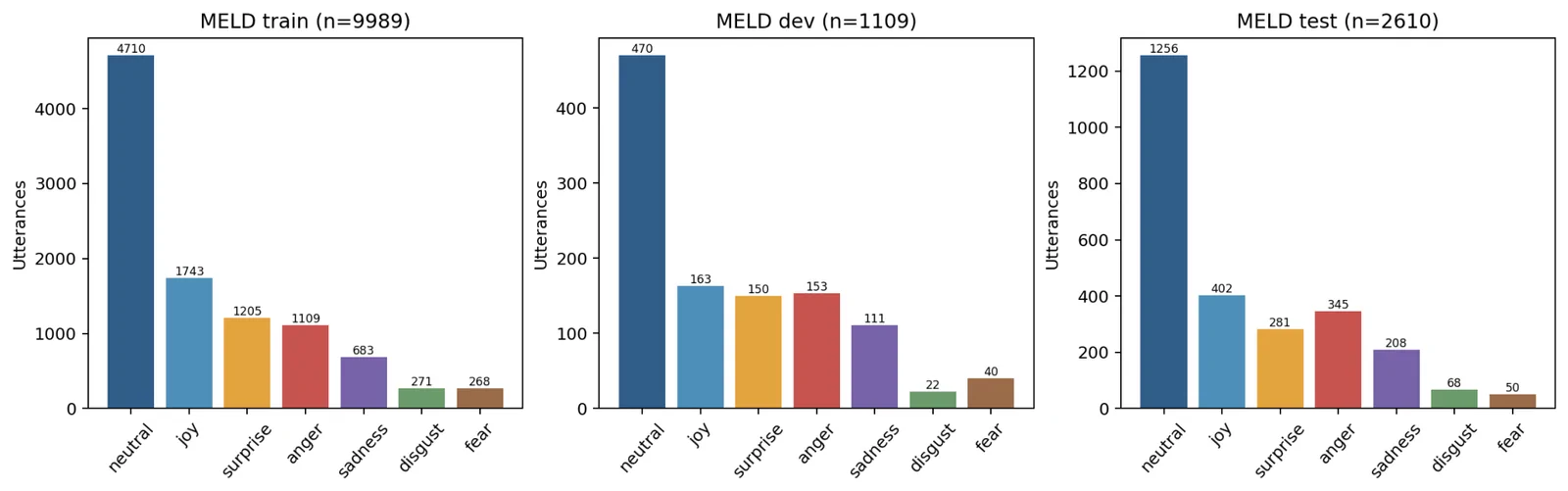
Official MELD emotion-label distribution for the train, development, and test splits.

The official test split has a 25.12:1 neutral-to-fear ratio (1256 vs. 50 utterances); across all three splits, the corresponding ratio is 17.98:1 (6436 vs. 358). Figure 4 visualizes this imbalance.

The retained checkpoint was evaluated twice under the same restored test-only procedure. Predicted labels, reference labels, and log-probabilities were identical element by element across the two evaluations. The restored pipeline produced WA 49.74%, weighted F1 46.27%, UA 30.69%, and macro F1 33.56% (Table 6). This result is close to, but not identical to, the historical 49.44% WA reported in the main comparison table; all class-wise analyses below use the restored 49.74% evaluation record.

**Figure 5:**
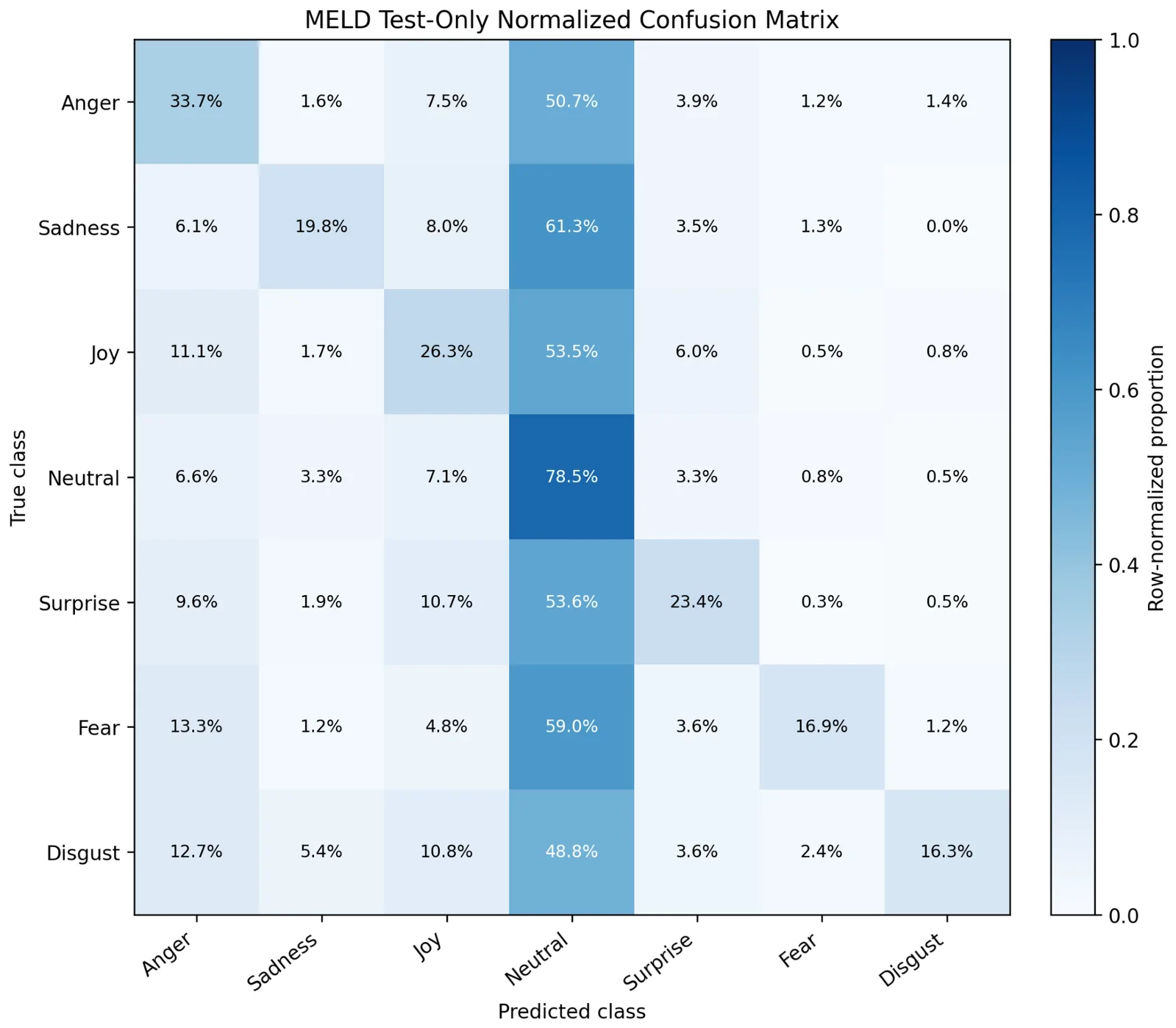
Row-normalized confusion matrix from the restored MELD test-only inference. Each row represents a reference class and sums to one.

Within the restored evaluation record, neutral constitutes 45.82% of the reference labels but 65.13% of model predictions. Class support has Spearman correlations of 0.93 with recall and 0.96 with F1, and the reported majority–minority macro-recall gap is 27.10 percentage points. These quantities indicate that errors are concentrated in lower-frequency classes and that predictions are strongly biased toward neutral. The row-normalized confusion matrix in Figure 5 is consistent with this pattern: 48.8–61.3% of disgust, anger, joy, surprise, sadness, and fear samples are assigned to neutral.

A 5000-replicate stratified bootstrap over the fixed restored-checkpoint predictions gives a 95% confidence interval of 48.42–51.05% for WA and 44.85–47.72% for weighted F1. These intervals quantify sample-level uncertainty for this fixed checkpoint; they do not represent variation across independently trained models. We use MELD’s original multi-party conversational recordings to analyze performance under its native acoustic variability and do not add artificial-noise experiments in this revision. Consequently, this analysis documents class imbalance and natural-condition errors but does not isolate the causal effect of individual noise factors.

### 6.4. Implementation-Consistent Interpretation of Positional Geometry

The positional analysis in Section 3.3 separates two questions that were conflated by a standalone positional-vector cosine comparison. First, $U_{\mathrm{index}}$ and $U_{\mathrm{phase}}$ quantify how uncertain the original token location remains after the stochastic FTS/FPE mapping. Second, $G_{pq}$ in Equation (25) describes the expected geometry of the implemented positional rotations by averaging over the actual candidate-index distributions. Neither quantity is a complete characterization of self-attention, because attention scores also depend on the learned, content-dependent query and key vectors.

Accordingly, we do not use pairwise cosine similarities between standalone APE, RoPE, and FPE vectors as evidence that one encoding “collapses” or preserves richer information. Any numerical comparison of positional geometry should be computed under matched sequence length, embedding dimension, numerical precision, and phase-collision tolerance. For stochastic FPE, it should additionally average over the FTS distribution $q_{pk}$ rather than use an unspecified single index realization. Classification behavior is examined independently through the available component-oriented comparisons above.

## 7. Discussion

### 7.1. Dataset-Dependent Observed Configurations

The selected configurations differ across the three evaluations. IEMOCAP-4 uses Fibonacci encoding in the log-mel branch and Fib+RoPE in the MFCC and Wav2Vec branches; IEMOCAP-6 replaces the Wav2Vec encoding with RoPE; and MELD uses APE for log-mel with Fib+RoPE in the other two branches. These differences may reflect dataset characteristics, optimization variability, model-capacity differences, or selection from many candidates. The present results do not isolate which explanation is responsible.

The conditional-entropy formulation in Section 3.3 does not predict these rankings; it quantifies position-recovery uncertainty under the implemented stochastic mapping. Accordingly, the main empirical conclusion is limited to the evaluated search: no single triplet is highest across all datasets, and Fibonacci-based configurations are not uniformly better than conventional ones. Capacity-matched multi-seed experiments are required before attributing branch-specific advantages to a particular positional mechanism.

### 7.2. Performance Differential Across Datasets

WA is higher on IEMOCAP-4/6 (74.21%/74.54%) than on the historical MELD evaluation (49.44%), although values across different label sets and protocols are not directly comparable. The document-backed analysis in Section 6.3 quantifies two relevant MELD properties. First, the official test split has a 25.12:1 neutral-to-fear imbalance. Second, the restored-checkpoint record shows 78.45% recall for neutral but only 16.27–26.35% for disgust, fear, sadness, surprise, and joy, with 65.13% of predictions assigned to neutral. The confusion matrix therefore localizes much of the observed error to minority-class samples being assigned to the majority class. These results support class imbalance as an important associated factor, while the absence of controlled acoustic perturbations prevents causal attribution to noise, overlap, or speaker transitions individually.

### 7.3. Representation-Specific Encoding Observations

Across the three highest-observed triplets, the MFCC branch uses Fib+RoPE, while the selected log-mel and Wav2Vec encodings vary. This repetition is noteworthy but is based on only three dataset/task settings and a single reported run per setting. We therefore describe it as an observed search pattern rather than a stable preference.

One hypothesis is that two-dimensional log-mel and MFCC grids and contextualized Wav2Vec sequences place different demands on position modeling. However, the current experiments do not separately manipulate locality, conversational noise, or temporal context, and therefore cannot verify this mechanism. Testing this hypothesis requires matched-capacity encoders and controlled perturbations of token ordering, noise, and sequence length.

The smaller observed increment from FTS on MELD (+0.37 percentage points over FPE-only) further indicates that the configuration-level difference is dataset-dependent in the available runs. Multi-seed estimates are needed to determine whether this small difference is distinguishable from training variability.

### 7.4. Scope of the Positional-Uncertainty Analysis

The implementation-consistent analysis establishes a limited but precise result: because the modulo-wrapped phase is a deterministic transformation of the sampled Fibonacci index, observing the phase cannot reveal more about the original token position than observing the complete index. The resulting conditional entropy quantifies positional recovery uncertainty, not representational richness, attention quality, or expected classification accuracy. The expected relation matrix further describes only the geometry of the positional rotations. We therefore base practical claims on the dataset-specific ablation results and treat the information-theoretic quantities as implementation diagnostics rather than evidence of causal superiority over APE or RoPE.

### 7.5. Limitations and Scope

This work has several limitations. First, the reported training results are based on a single random seed; fixed-checkpoint resampling cannot replace independent multi-seed training, confidence intervals across runs, and significance testing. Second, the conventional and Fibonacci configurations differ in attention-head count, so the current comparisons do not isolate positional encoding from model capacity. Third, optimization exposure is not fully matched: RoPE is evaluated in the common first-stage search but does not receive the same second-stage learning-rate refinement budget as the shortlisted configurations and APE/ALiBi baselines. Fourth, the model uses fixed-length inputs, and generalization to variable-length utterances is not evaluated. Fifth, evaluation is limited to English datasets and does not establish multilingual or cross-cultural generalization. Sixth, the MELD analysis quantifies class imbalance and errors under the dataset’s native conversational conditions but does not include artificial-noise or controlled-imbalance experiments, so individual acoustic causes remain unresolved. Seventh, the official MELD distribution and restored-checkpoint class-wise record use different counting bases (2610 raw test utterances versus 3808 processed evaluation samples) and are intentionally reported separately. Eighth, the positional-uncertainty quantities measure location recoverability and rotation geometry rather than complete content-dependent attention behavior; their values depend on the token grid, candidate sets, embedding dimension, numerical precision, and collision tolerance. Ninth, FTS has not been compared with alternative structured index assignments, such as zig-zag scans, Hilbert curves, or other space-filling curves. Finally, cross-study comparisons remain incompletely controlled because preprocessing, features, model capacity, and evaluation protocols differ.

## 8. Conclusions

This paper presents FPE and FTS for candidate-based Fibonacci phase assignment in a multi-representation acoustic SER model. Across the evaluated exploratory search, the highest-observed configurations reach 74.21% WA on IEMOCAP-4, 74.54% on IEMOCAP-6, and 49.44% on MELD, with different triplets selected for the three settings. The implementation-consistent conditional-entropy formulation quantifies positional recovery uncertainty introduced by candidate sampling and modulo-wrapped phases; it does not establish richer representations or superior attention. Available component runs document larger configuration-level differences between FPE-only and FPE+FTS on IEMOCAP than on MELD. The restored MELD analysis further identifies strong prediction bias toward neutral and substantially lower recall for minority emotions, while fixed-checkpoint bootstrap intervals quantify sample-level uncertainty. Future work will address variable-length processing, multilingual evaluation, controlled noise and imbalance experiments, and comparisons with alternative two-dimensional index-assignment strategies.

## Figures and Tables

**Table 1:** Performance comparison of prior SER models and STRE-Former on IEMOCAP and MELD. Bold values indicate the best results for each metric. “Mod.” denotes source modality: A = audio only, T+A = text and audio, and LLM = large language model. For STRE-Former, the highest-observed refined configuration is reported for each dataset. Direct comparison is limited by differences in feature pipelines, data augmentation, model capacity, and evaluation protocols across studies.

Model	Mod.	IEMOCAP(4)			IEMOCAP(6)			MELD	
		WA	UA	WF1	WA	UA	WF1	WA	WF1
CNN–LSTM [4]	A	62.9	57.1	59.5	51.3	46.5	47.3	–	–
LSTM-Attn [16]	A	56.5	56.7	56.5	43.4	43.6	43.4	–	–
MAEC [54]	A	68.17	69.28	–	–	–	–	42.07	–
SpeechFormer [19]	A	62.9	64.5	–	–	–	–	41.9	–
DWFormer [20]	A	72.3	73.9	–	–	–	–	–	**48.5**
SpeechFormer++ [55]	A	70.5	71.5	70.7	–	–	–	**51.0**	47.0
CubicKD [40]	A	–	–	–	63.32	64.29	–	–	–
TENT [56]	A	72.43	73.88	–	–	–	–	–	45.1
ENT [56]	A	71.84	72.37	–	–	–	–	–	46.3
AA-SLLM [53]	LLM	–	85.50	–	–	–	–	–	55.23
SpeechCueLLM [52]	T+A	–	–	–	–	–	72.6	–	67.6
STRE-Former (ours)	A	**74.21**	68.31	**73.96**	**74.54**	**70.48**	**74.29**	49.44	45.87

**Table 2:** Configuration-level computational efficiency comparison. Bold values indicate the optimal result for each metric. The Fibonacci and conventional baselines differ in attention-head count. All measurements use an NVIDIA GeForce RTX 4090, batch size 16, 50 warm-up iterations, and 200 measurement runs.

Configuration	Heads	Params (M)	FLOPs (G)	Latency (ms)	GPU Mem (MB)
Ours (F-FR-FR)	1	**161.79**	**22.94**	13.87	**1424.7**
APE	4	162.90	26.78	**9.89**	1425.8
ALiBi	4	162.90	26.78	**8.71**	1424.8

**Table 3:** Highest-observed refined positional-encoding configurations on each dataset. These values are selected from multiple configurations and should not be interpreted as stable rankings without repeated runs.

Dataset	Spec	MFCC	Wav2Vec	WA (%)	WF1 (%)	UA (%)	UWF1 (%)
IEMOCAP-4	Fib	Fib+RoPE	Fib+RoPE	74.21	73.96	68.31	72.07
IEMOCAP-6	Fib	Fib+RoPE	RoPE	74.54	74.29	70.48	73.49
MELD	APE	Fib+RoPE	Fib+RoPE	49.44	45.87	30.34	33.13

**Table 4:** Available configuration-level comparison of Fibonacci components (WA, %). The No-Fibonacci reference uses APE in all three branches; FPE-only disables FTS candidate sampling; FPE+FTS denotes the highest-observed full configuration. The No-Fibonacci comparison is not capacity-matched.

Dataset	No Fibonacci	FPE-Only	FPE+FTS	ΔFTS
IEMOCAP-4	69.73	70.11	74.21	+4.10
IEMOCAP-6	65.53	67.30	74.54	+7.24
MELD	46.50	49.07	49.44	+0.37

**Table 5:** Official MELD emotion-label distribution by split. Test proportion is calculated over the 2610 official test utterances.

Emotion	Train	Development	Test	Test Proportion (%)
Neutral	4710	470	1256	48.12
Joy	1743	163	402	15.40
Surprise	1205	150	281	10.77
Anger	1109	153	345	13.22
Sadness	683	111	208	7.97
Disgust	271	22	68	2.61
Fear	268	40	50	1.92
Total	9989	1109	2610	100.00

**Table 6:** Overall MELD metrics from the restored-checkpoint evaluation (%).

WA	Weighted F1	UA	Macro F1
49.74	46.27	30.69	33.56

**Table 7:** Class-wise MELD results from the 3808 samples processed by the restored-checkpoint evaluation.

Emotion	Support	Precision (%)	Recall (%)	F1 (%)
Neutral	1745	55.20	78.45	64.80
Joy	630	40.10	26.35	31.80
Surprise	364	38.64	23.35	29.11
Anger	507	38.60	33.73	36.00
Sadness	313	40.00	19.81	26.50
Disgust	166	54.00	16.27	25.00
Fear	83	30.43	16.87	21.71

## Data Availability

The IEMOCAP dataset is available from the USC SAIL Lab at https://sail.usc.edu/iemocap/, subject to the dataset access conditions (accessed on 1 July 2026). The MELD dataset is available at https://affective-meld.github.io/ (accessed on 1 July 2026). The code and processed experimental configurations will be released in a public repository upon acceptance, and the repository link will be provided in the final version.
